# Mental Health of Emergency Department Healthcare Workers During COVID-19 in Brooklyn, New York

**DOI:** 10.18103/mra.v10i7.2903

**Published:** 2022-07-31

**Authors:** Deborah R Gustafson, Recai Yucel, Samuel J Apple, Gianna Cirrone, Haoyuan Gao, Aaron J Huang, Xinrui Ma, Ayesha Saad, Jeremy Wilson, Sarah Kabariti, Sergey Motov

**Affiliations:** 1 Department of Neurology, State University of New York Downstate Health Sciences University, Brooklyn, New York; 2 Department of Biostatistics, Temple University, Philadelphia, Pennsylvania; 3 State University of New York Downstate Health Sciences University, College of Medicine, Brooklyn, New York; 4 Department of Emergency Medicine, Maimonides Medical Center, Brooklyn, New York; 5 Department of Emergency Medicine, State University of New York Downstate Health Sciences University, Brooklyn New York

## Abstract

**Background.:**

Maintaining good mental health among Emergency Department healthcare workers (ED HCW) is paramount to well-functioning healthcare. We measured mental health and COVID-19 symptoms in ED HCW at a COVID-19 epicenter.

**Methods.:**

A cross-sectional, convenience sample of adult (≥18 years) ED HCW in Brooklyn, New York, USA, who were employed at ≥50% of a full-time effort, was surveyed September–December, 2020 with reference period March-May 2020. An anonymous email-distributed survey assessed gender, age, race, healthcare worker status (clinical versus non-clinical), SARS-CoV-2 testing, number of people to talk to, COVID-19-related home problems, mental health care interruption during COVID-19, loneliness, and survey date. Outcomes included symptoms of depression, psychological distress, perceived stress, post-traumatic stress disorder (PTSD), anxiety, and resilience measured using validated scales.

**Results.:**

Of 774 HCW, 247 (31.9%) responded (mean age 38.2±10.8 years; 59.4% White; 52.5% men; 80.1% clinical; 61.6% SARS-CoV-2 tested). Average mental health scores were significantly higher among clinical vs non-clinical HCW (P’s<0.0001–0.019). The proportion reporting a clinically-relevant psychological distress symptom burden was higher among clinical vs non-clinical HCW (35.8% vs 13.8%, p=0.019); and suggested for depression (53.9% clinical vs 35.7% non-clinical, p=0.072); perceived stress (63.6% clinical vs 44.8% non-clinical, p=0.053); and PTSD (18.2% clinical vs 3.6% non-clinical, p=0.064). Compared to non-clinical staff, Medical Doctors and Doctors of Osteopathy reported 4.8-fold higher multivariable-adjusted odds of clinically-relevant perceived stress (95%CI 1.8–12.9, p=0.002); Emergency Medical Technicians reported 15.5-fold higher multivariable-adjusted odds of clinically-relevant PTSD (95%CI 1.6–150.4, p=0.018). Increasing age, number of COVID-19-related home problems and people to talk to, loneliness and mental health care interruption were adversely associated with mental health; being male and SARS-CoV-2 testing were beneficial.

**Conclusions.:**

COVID-19-related mental health burden was high among ED HCW in Brooklyn. Mental health support services are essential for ED HCW.

## Introduction

Healthcare workers (HCW) around the world worked in the vortex of CoronaVIrus Disease (COVID-19) in 2020–2022.^1,2^ New York City was the epicenter of the COVID-19 outbreak in the United States from February 29–June 1, 2020. During this period there were ~203,792 (2263/100,000) laboratory-confirmed COVID-19 cases reported to the New York City Department of Health and Mental Hygiene.^3^ Of these, 56,548 (2104 per 100,000) were Brooklyn residents, among whom there were 15,125 (26.7%) hospitalizations (556 per 100,000) and 5563 (9.8%) deaths (205 per 100,000).^3^

Emergency Department (ED) HCW, a large workforce,^4,5^ dealt with a high proportion of COVID-19 patients due to the acute nature of this highly contagious and often severe disease. Serious tolls on ED HCW mental health included higher than expected symptoms of depression, anxiety, perceived stress, distress, post-traumatic stress disorder (PTSD), and even suicide.^2,6^ This was exacerbated by a high risk of personally contracting the infection and/or passing it to family members and co-workers, excessive levels of pressure from working long shifts, and witnessing excess mortality. Early on in the pandemic, there was inadequate testing for the Severe Acute Respiratory Syndrome Coronavirus (SARS-CoV-2), limited treatment options for COVID-19, and lack of personal protective equipment. As a result, many HCW chose to transition from acute to chronic care positions or non-clinical places of employment.^7^ COVID-19 is the third large-scale infectious disease outbreak in the 21^st^ century, occurring <20 years after the 2003 SARS outbreak. It is reasonable to hypothesize that this scenario will likely occur again and may again affect HCW who are employed today.

While numerous studies have measured and reported that mental health among HCW was poor during the COVID-19 pandemic,^8–13^ many of these studies did not use validated measures of mental health, used mental health measures that were inappropriate for the culture or language, or used survey distribution and/or completion methods that may have exacerbated adverse mental health symptoms via causing undue stress on the respondent.^14^ Nonetheless, the majority of studies have reported poor mental health among HCW during COVID-19.

Brooklyn is one of five boroughs comprising New York City, and has a number of characteristics that contributed to its emergence as a COVID-19 epicenter, with rapid spread of SARS-CoV-2 and subsequent severe impacts on HCW during early-to mid-2020. In 2022 (similar to 2020), Brooklyn has a population of 2.6 million people (highest census among the five boroughs), with a very high population density of 14,182 per square kilometer (36,732 people per square mile).^15^ This contributes to a very high level of built environment, i.e., man-made or modified structures that provide people with living, working, and recreational spaces, but generally lacking green and open spaces.^16^ Most recent census data from 2010, indicated that Brooklyn residents were 49.5% White (35.8% non-Hispanic), 35.8% African American, 11.3% Asian, and 3.4% Other; 19.8% self-identified as Hispanic or Latino of any race. In addition, 38% were foreign-born.^15^ Brooklyn HCW serve this highly heterogeneous community, mixed not only in race and ethnicity, but also in socioeconomic status, health equity, educational attainment, gender, religion, prevalent co-infections (HIV, HCV) and other factors. Against a background of high population density and built environment, it is a useful population to assess in order to measure the mental health of ED HCW during this critical time period.^17^

We conducted an email-distributed survey among ED HCW at a medical center that is a major healthcare provider in Brooklyn, one which sees over 120,000 sociodemographically, racially and ethnically diverse patients annually. This survey retrospectively assessed mental health using valid, commonly used, and appropriate scales to measure symptoms of depression, psychological distress, perceived stress, PTSD, anxiety and resilience during the peak of the COVID-19 pandemic, between March 2020 and June 2020. COVID-19 symptoms and testing while working in the ED were also assessed, as were sociodemographic factors. During this peak, approximately 1–2% of all COVID-19-related mortality in the United States occurred at this one institution. This report fills a knowledge gap in the assessment of mental health of an under-represented, at-risk population that will guide future observational and interventional research efforts.

## Methods

### Study Design and Setting

A cross-sectional, email-distributed survey of ED HCW in Brooklyn, New York, was conducted between September 8, 2020 and December 31, 2020. The reference period was March 2020-June 2020, when Brooklyn experienced the peak impact of COVID-19.

### Study Participants

Participant eligibility criteria included: adult (≥18 years) ED HCW at Maimonides Medical Center who were employed at ≥50% of a full-time effort. Employment status eligibility was determined via the ED employee roster. Eligible participants were emailed a description and link to the survey. The link was valid until the survey was completed. Participants were emailed a weekly reminder from the original e-mail date. If an out of office reply was received, the email was sent again one month later. If the email bounced back as unrecognized, that person was deemed ineligible. Participants could choose to answer any or all survey items. Upon receipt of survey results, data were checked.

Study data were collected and managed using REDCap (version 9.5.3) electronic data capture tools hosted at Maimonides Medical Center.^18,19^ REDCap (Research Electronic Data Capture) is a secure, web-based software platform designed to support data capture for research studies, providing 1) an intuitive interface for validated data capture; 2) audit trails for tracking data manipulation and export procedures; 3) automated export procedures for seamless data downloads to common statistical packages; and 4) procedures for data integration and interoperability with external sources. This protocol was approved by the Maimonides Medical Center institutional review board, and deemed exempt because the survey was anonymous and did not contain personal identifiers.

### Data Source and Collection

The Mental Health in HCW pilot survey included questions about sociodemographic factors, validated mental health scales, and components of the baseline MACS/WIHS Combined Cohort Study COVID-19 Survey available at https://statepi.jhsph.edu/mwccs/data-collection-forms/. ^20^ Prior to being sent for production, the pilot survey was extensively overviewed multiple times among the investigator team.

Sociodemographic factors included age, sex/gender, race/ethnicity (African American/Black, Asian, Hispanic, White, Other), and HCW occupation categorized as clinical (direct contact with patients: Medical Doctors, Doctors of Osteopathic Medicine, Registered Nurses, Nurse Practitioners, Physician Assistants, Emergency Medical Technicians, patient care technicians, mental health workers, and scribes) and non-clinical (ancillary and supporting staff: clinical pharmacists, mental health workers, registration clerks, research personnel, scribes, residency administrators, departmental administrators, and other).

The impact of COVID-19 was measured as 13 COVID-19 symptoms, SARS-CoV-2 infection testing (yes/no) and status (infected, not infected, test result unknown), hospitalization for COVID-19, pharmaceutical treatment for COVID-19, and recovery status. In addition, steps taken to reduce infection such as staying at home, social distancing, self-quarantine, and making changes in daily routine were queried. We also assessed increased vs decreased use of over-the-counter and prescription pain medications for migraine or body aches during COVID-19.

A variety of sociobehavioral factors were evaluated, including changes in physical, emotional and sexual abuse; interruptions in mental healthcare; interruptions in substance use addiction services; problems accessing healthcare; loneliness via the Loneliness Brief Form;^21^ number of people to talk to (range: 0- ≥6); satisfaction with number of people to talk to; and presence of COVID-19 created problems at home such as job loss, receiving unemployment benefits, loss of childcare, loss of other resources & financial support, change or loss in health insurance, loss of housing or becoming homeless, loss of health insurance, gain of emergency health coverage, and difficulty paying for basic needs.

### Study Outcomes

Mental health scales for the primary outcomes included the Center for Epidemiologic Studies – Depression Scale (CES-D),^22^ the Kessler Psychological Distress Scale (K10),^23^ Perceived Stress Scale-4 (PSS-4),^21^ and PTSD Checklist for DSM-5 (PCL-5).^24^ The 2-item PROMIS® scale^25^ for anxiety and 3-item Brief Resilient Coping Scale^26^ were also included.

The presence and severity of depressive symptoms were assessed using the 10-item CES-D.^27^ The CES-D range was 0–30 and a cutoff of ≥10 was deemed to be a clinically-relevant depressive symptom burden. The K10 measured psychological distress using a 10-item scale (range 10–50). Clinical interpretation of K10 score is: <20, likely to be well; 20–24, likely to have a mild mental disorder: 25–29, likely to have moderate mental disorder; and ≥30, likely to have a severe mental disorder. A cutoff of ≥20 was deemed to be clinically-relevant psychological distress.^23^ Perceived stress was assessed using the PSS-4 (range 0–16).^28^ A clinically-relevant cutoff of ≥6 is an accepted normative value.^29^ PTSD was assessed using the PCL-5, a 20-item psychometrically sound measure that assessed the presence and severity of PTSD symptoms (range 0–80).^24^ A PCL-5 score of ≥32 was deemed clinically-relevant PTSD.^30^ The 2-item PROMIS® Anxiety Short Form was used to assess anxiety (range 0–6).^25^

In contrast, the ability to manage stress in an adaptive manner was measured using a 3-item Brief Resilient Coping Scale (range 0–15).^26^ Using this metric, participants were asked to reflect on ways in which they were able to cope with stressful events in their lives.

### Data Analysis

The survey response rate was calculated as the number of completed surveys returned from recipients’ emails divided by the number of employees with successful email contact (i.e., no ‘bounce backs’ or out of office replies). Multiple attempts to contact individual employees did not add to the denominator. The denominators for all descriptive variables and individual mental health outcomes differed slightly as we used any and all data collected.

Descriptive data analyses included frequencies and percentages of categorical variables (e.g., number (%) of COVID-19 symptoms among those who replied), as well as means and standard deviations of continuous variables (e.g., PCL-5 score of 0–80). Spearman and Pearson correlation coefficients were calculated among continuous variables. Dichotomized outcome scores were examined in relation to categorical predictors using Chi-square analyses. Student *t*-tests were used to evaluate mean continuous outcomes scores between clinical and non-clinical HCW groups.

Prediction models included both continuous and dichotomous outcomes of depression, psychological distress, perceived stress, and PTSD symptoms and resilience. Linear regression models, with output of beta-coefficients and 95%CI, were fitted for continuous mental health outcome scores.

Assumptions of normal distributions of continuous variables were assessed and met. Logistic regression analyses were fitted for dichotomous mental health outcomes to estimate adjusted Odds Ratio (OR) estimates along with 95% Confidence Interval (95% CI). The primary predictor in all models was clinical versus non-clinical HCW. Covariates of interest included gender, age, race, SARS-CoV-2 testing (yes/no) and status (positive/negative/unknown), number of people to talk to, COVID-19-related home problems, mental health care disruption during COVID-19 (yes/no), Loneliness Brief Form score, and survey date. Covariates included in final multivariable regression models were significant at p<0.10 in univariate models. The presence of collinearity among independent variables was checked and confirmed not present, thus all could be included in the same regression models.

The data analyses for this paper were generated using SAS software, Version 9.4, Copyright © 2013, SAS Institute Inc. Cary, NC, USA. All results were considered significant at a two-tailed p<0.05.

## Results

### General participant characteristics

The survey was sent to 774 unique ED healthcare worker e-mails, of whom 247 (31.9%) completed the survey. Of these, 84.6% (N=208) were clinical HCW; and 15.4% (N=38) were non-clinical HCW. Of clinical HCW, 30.5% were physicians (Medical Doctors or Doctors of Osteopathy); 23.5% Registered Nurses, Nurse Practitioners, or Physicians’ Assistants; 25.2% Emergency Medical Technicians; and 5.3% other. Approximately half of the participants were women and half men; majority (56.8%) White; and average age 38.2±10.8 years with the age group 25–34 years containing the highest proportion of participants (40.3%) compared to other 10-year age groups (Table 1).

### COVID-19 characteristics

Table 2 shows self-reported COVID-19 protective measures, symptoms, care seeking, SARS-CoV-2 testing and treatments reported by participants. Some type of protective measure against SARS-CoV-2 infection was adopted by >99% of both clinical and non-clinical HCW. The most common behavior was social distancing (reported by 93.2% of all participants). While almost 40% (N=96) reported experiencing >1 COVID-19 symptom, only 17.3% (N=43) sought care and 2.5% were hospitalized for COVID-19. Testing for SARS-CoV-2 was reported by 63.2% (N=156). At the time of the study PCR-based testing was not universally available, even for HCW. Of those tested, 18.2% (N=28) reported a positive test result. There were 54 ED HCW who reported taking COVID-19 medications (Table 2). Increased use of over-the-counter pain medications was reported by 23% of clinical HCW, corresponding to the most common symptoms reported: muscle aches (34.6%), fever (32.1%), and headache (30.5%).

### Mental health outcomes

Average mental health scores and percentages of those with clinically-relevant mental health burden by healthcare worker status are shown in Table 3. Multivariable-adjusted models predicting mental health outcomes are presented in Tables 4 and 5. Notably, clinical HCW exhibited 1.3 to 2.1 times higher mean scores than non-clinical HCW (p’s<0.05) on scales of depression (CES-D), psychological distress (K10), perceived stress (PSS-4), and PTSD (PCL-5).

Clinically-relevant depressive symptoms were reported by among 51.6% (53.9% of clinical HCW *vs* 35.7% of non-clinical HCW, p=0.072). We observed 8% higher odds of clinically-relevant depressive symptoms with increasing age (OR 1.08, 95%CI 1.04–1.12), a 49% increase with increasing COVID-19-related home problems (OR 1.49, 95%CI 1.02–2.17), and a 2-fold increase with increasing loneliness score (OR 2.09, 95%CI 1.63–2.67). Similarly, increasing CES-D scores were observed with increasing age (β=0.12, p=0.010), number of COVID-19-related home problems (β=0.99, p=0.035), and loneliness score (β=2.17, p<0.0001).

Clinically-relevant psychological distress (K10 score≥20) was observed among 35.8% clinical vs 13.8% non-clinical HCW (p=0.019). There was a 2-fold higher odds of clinically-relevant psychological distress with each point increase in loneliness score (OR 2.20, 95%CI 1.69–2.86), and >6-fold higher odds with having >0 people to talk to. Testing negative (−) for SARS-CoV-2 was protective for psychological distress (OR 0.40, 95%CI 0.17–0.94). A higher K10 score was associated with being an emergency medical technician (β=3.69, p=0.007), increasing loneliness score (β=2.20, p <0.0001), mental health care interruption (β=3.45, p=0.036), and having ≥4 people to talk to (β=3.67, p=0.018). Lower K10 scores were observed among men (β=1.98, p=0.043).

Among clinical HCW, 63.6% reported clinically-relevant perceived stress compared to 44.8% of non-clinical HCW (p=0.053). Physicians (MD/DO) had >4.5-fold higher odds of clinically-relevant perceived stress (OR 4.57, 95%CI 1.73–12.04) compared to non-clinical ED HCW; those reporting increasing COVID-19-related home problems, a 2-fold higher odds (OR 2.01, 95%CI 1.34–3.02); and with increasing loneliness brief form score, >1.5-fold higher odds (OR 1.68, 95%CI 1.34–2.10). Testing positive for SARS-CoV-2 was protective for clinically-relevant perceived stress (OR 0.26, 95%CI 0.08–0.84) compared to not being tested. Testing negative was also suggested to be protective (p=0.064). When evaluated as a continuous outcome, being tested, whether the result was negative (β=−1.11, p=0.008) or positive (β=−1.65, p=0.012) was protective for perceived stress. Lower perceived stress scores was also observed in men (β=−0.87, p=0.035). Increasing perceived stress was observed among MD/DO (β=1.43, p=0.010), and with increasing number of COVID-19-related home problems (β=0.69, p=0.001), ≥4 people to talk to (β=1.44, p=0.028), and increasing loneliness brief form score (β=0.62, p<0.0001).

Clinically-relevant PTSD was found among 16.3% of HCW (18.2% of clinical *vs* 3.6% of non-clinical HCW, p=0.064). ED technicians had a 15.5-fold higher odds of clinically-relevant PTSD symptoms (OR 15.49, 95%CI 1.60–150.37) compared to non-clinical HCW. However, this should be cautiously interpreted due to relatively higher standard error. Those reporting increasing loneliness scores also had higher odds of PTSD (OR 1.69, 95%CI 1.28–2.23). Higher PTSD scores were observed among emergency medical technicians (β=7.50, p=0.012), and with an increasing number of COVID-19-related home problems (β=2.13, p=0.04), increasing loneliness score (β=4.10, p<0.0001), and ≥4 people to talk to (β=6.97, p=0.038); lower PTSD scores were observed for men (β=−4.31, p=0.048).

Anxiety measured by mean responses to ‘My worries overwhelmed me’ and ‘I felt uneasy’ (range 0–6) was 1 point higher among clinical (mean score 2.3±2.2) versus nonclinical (mean 1.3±1.6) HCW (p=0.019).

The mean Brief Resilient Coping Scale score was 11.1±2.5 (range 0–15). Lower resilience was observed with having ≥4 people to talk to compared to no one (β=−1.52, p=0.009). Higher resilience was observed among those who tested negative for SARS-CoV-2 compared to those not tested (β=0.81, p=0.027). There is no clinically-relevant cutoff for resilience using the 3-item Brief Resilient Coping Scale.

## Discussion

Our data illustrate the mental health burden among ED HCW in a heavily impacted community during the peak of the COVID-19 pandemic in 2020. We observed >50% of ED HCW had clinically-relevant depressive and perceived stress symptom burdens, 30% had psychological distress and almost 20% had PTSD. Prevalence of clinically-relevant mental health symptom burdens was higher among clinical ED HCW, particularly emergency medical technicians and Medical Doctors or Doctors of Osteopathy, compared to non-clinical ED HCW. Loneliness and having more people to talk to were also related to worse mental health. Testing status (whether positive or negative), and being a man were related to better mental health outcomes.

There are a plethora of published reports and reviews globally, of high mental health burden among HCW during COVID-19. Some of these reports are referenced herein. Literature reviews and original research articles support our findings, the importance of this issue, and the urgency to address it.^1,2,31,32,33,34^ As of June 23, 2022, there have been 2.57 million COVID-19 cases and 40,662 COVID-19 deaths in New York City. The borough of Brooklyn (Kings County) alone has experienced 711,000 COVID-19 cases (27.7% of all in New York City), and 9754 deaths (24.0% of all in New York City).^35^ Accordingly, there have been several New York City healthcare institution responses to this pandemic;^17,36,37^ and guidance on the vital role of psychiatric emergency clinicians has been published by the American Association for Emergency Psychiatry (AAEP).^38^

Unfavorable mental health symptoms among ‘frontline’ HCW including those working in the ED,^39,40^ intensive care units,^41^ and other HCW who have been in direct contact with patients with COVID-19 are reported to be common.^11,31,42–51^ The prevalence of clinically-relevant depressive, perceived stress, PTSD, and anxiety symptoms were higher among Brooklyn HCW compared to other published reports,^52–56^ and similar to those reported among ED HCW, specifically.^11,39^ We are one of the few published studies to compare clinical and non-clinical ED HCW since most published studies focus on clinical and/or frontline HCW only. In addition, sex differences have often not been addressed. Our data concur with others that women HCW reported worse mental health compared to men.^52,57^

Our data also suggest that both loneliness and a having a higher number of people to talk to were related to worse mental health. While this may seem contradictory, other studies have published similar findings.^1,31,40,51,57^ Having more people to talk to may not have been advantageous given social distancing mandates during COVID-19. Lower resilience scores were also observed among those with ≥4 people to talk to compared to no one, which also may have contributed to poorer mental health. Loneliness may have been more prevalent since almost 67% of ED HCW reported staying home as much as possible as a protective measure against SARS-CoV-2 infection. In addition, most social gathering places, such as restaurants and churches, were closed. The combination of having too many people to talk to and loneliness being associated with poorer mental health illustrates the complex social effects of COVID-19.

The situation for United States healthcare is dire due to overall changes in patient care delivery, which speaks to the need to consider both clinician and patient safety. In April 2021, a survey was conducted by the Washington Post and Kaiser Family Foundation of 1327 HCW who had direct contact with patients and their bodily fluids in hospitals, doctors’ offices, outpatient clinics, nursing homes, assisted care facilities, and those working in home health care. At that time, 29% of HCW reported they had ‘considered leaving the field’.^58^ In addition, worry or stress due to COVID-19 caused 47% of HCW to have trouble sleeping or to be sleeping too much, 31% reported frequent headaches or stomach aches, and 16% increased drug or alcohol use; 56% experienced any of these issues.^7^ At least 40% of these frontline HCW stated that the pandemic had negatively impacted their physical health (49%) and relationships with family members (42%) and coworkers (41%).^7^

Our study of mental health in HCW had many strengths compared to other published studies. We responded in a timely manner to the COVID-19 epidemic in central Brooklyn ED HCW, one of the hardest hit workforce communities in New York City. We used an efficient, easily-administered, and inexpensive email-administered REDCap survey. Participant responses were anonymous. We addressed several potential confounders and effect modifiers including age, sex and gender, race and ethnicity, HCW occupation, SARS-CoV-2 testing and precautionary behaviors. We compared clinical and non-clinical HCW. The proportion responding was 31.9%, which is laudable given the survey administration method and the short time period (4 months) of survey administration. We had ethical review board approval, which is often not reported in published studies.^59^ Finally, these data form the critical basis for future research of mental health in HCW.

### Limitations

As is common with anonymous, self-reported, retrospective survey-based methods, there were limitations. Some of these limitations include the following. 1) Data collected during this study were not checked against medical or psychiatric records. 2) The study design was cross-sectional and there were no historical mental health assessments for pre-pandemic comparisons. Cross-sectional studies cannot address causal associations. While some studies have conducted mediation analyses within cross-sectional study designs,^60^ we did not use this analytic approach since it relies on assumptions of directionality and temporality of associations that may be incorrect. It is therefore uncertain as to whether COVID-19 triggered incident adverse mental health symptoms, exacerbated existing or preclinical mental health symptom burden, or whether poor mental health existed already among HCW and remained during the pandemic.^14^ There are a small number of longitudinal studies among HCW reporting levels of, for example, anxiety^61^ and psychological distress,^62^ which reported that they increased within 6 months from the beginning of the pandemic. However, published longitudinal studies are rare and have a short duration of follow-up. This can be addressed in future studies. 3) Our survey was limited to ED HCW, and may not be generalizable to non-ED HCW. 4) Non-respondents may have differed systematically (e.g., too stressed, concurrent caregiver roles, diagnosed and/or severe COVID-19) from respondents. 5) Given the retrospective nature of the study, there was the possibility of recall bias and errors in reporting. However, survey completion date was included as a covariate and not related to any mental health outcome. 6) The study was conducted electronically and without face-to-face contact, therefore clarifications could not be made if a survey item was misunderstood. However, we extensively pilot-tested this survey among the research team and used well-known, validated scales and questions that have been used in similar clinical and population-based data collection efforts. 7) COVID-19 symptoms data were descriptive and could not be solely attributed to COVID-19 given other illnesses with these symptoms. 8) We did not gather data on socioeconomic status, household size, number of years in practice among clinical HCW, COVID-19-related stressors at home (e.g., lack of childcare), access to personal protective equipment, fears regarding transmission to family members, witnessing excessive death, and other factors. Thus, we cannot definitively say that the mental health burden observed in study participants was fully attributable to their ED HCW status during the COVID-19 pandemic. Finally, 9) for certain occupations, particularly among non-clinical HCW, our sample size does not allow stratified analyses. Despite these limitations, we achieved our pilot study goals and have collected critical first-ever preliminary and foundational data to take next research steps in our local community.

## Conclusions

Describing associations between ED HCW status and mental health during the COVID-19 outbreak is crucial in guiding policies and interventions to maintain mental well-being in this occupational group and prevent long-lasting sequelae. The mental health status of HCW is paramount to maintaining optimally-functioning healthcare systems.

## Figures and Tables

**Table 1.** Demographic and occupational characteristics of clinical and non-clinical ED HCW in Brooklyn, New York

Characteristic	Total (N, %)	Clinical HCW (N, % of clinical)	Non-Clinical HCW (N, % of non-clinical)
**Gender**			
Female	11 8 (47.8%)	104 (48.6%)	12 (41.4%)
Male	127 (51.4%)	108 (50.5%)	17 (58.6%)
Other	2 (0.8%)	2 (0.9%)	0
**Age Group (years)**			
18 – 24	11 (4.5%)	9 (4.3%)	2 (6.9%)
25 – 34	98 (40.3%)	87 (41.4%)	11 (37.9%)
35 – 44	65 (26.8%)	57 (27.1%)	6 (20.7%)
45 – 54	43 (17.7%)	35 (16.7%)	7 (24.1%)
55 – 64	22 (9.1%)	18 (8.6%)	3 (10.3%)
>= 65	4 (1.7%)	4 (1.9%)	0
**Race/Ethnicity**			
White	138 (56.8%)	122 (58.1%)	12 (41.4%)
Black	31 (12.8%)	25 (11.9%)	6 (20.7%)
Asian	42 (17.3%)	37 (17.6%)	5 (17.2%)
Other	32 (13.2%)	26 (12.4%)	6 (20.7%)
**Occupation**			
**Clinical HCW**			
Medical Doctor/Doctor of Osteopathy	75 (30.5%)	75 (35.1%)	0
Nurse			
Registered Nurse	52 (21.1%)	52 (24.3%)	0
Nurse Practitioner	4 (1.6%)	4 (1.9%)	0
Physician Assistant	2 (0.8%)	2 (0.9%)	0
Emergency Medical Technician	62 (25.2%)	62 (29%)	0
Other			
Patient Care Technician	8 (3.3%)	8 (3.7%)	0
Mental Health Worker	2 (0.8%)	2 (0.9%)	0
Scribe	3 (1.2%)	3 (1.4%)	0
**Non-clinical HCW**			
Pharmacy	3 (1.2%)	0	3 (10.3%)
Register/Clerk	7 (2.9%)	0	7 (24.1%)
Research Personnel	10 (4.1%)	0	10 (34.5%)
Residency Administrator	2 (0.8%)	0	2 (7%)
Departmental Administrator	2 (0.8%)	0	2 (7%)
**Other**	14 (5.7%)	6 (2.8%)	5 (17.2%)

**Table 2.** Self-reported COVID-19 protective measures, symptoms, care seeking, SARS-CoV-2 testing and treatments among clinical and non-clinical ED HCW in Brooklyn, New York

	Total N (%)	Clinical HCW N (% of clinical)	Non-Clinical HCW (N (% of nonclinical)
**Protective measures against SARS-CoV-2 infection**			
Staying home as much as possible	165 (66.5%)	139 (65.3%)	22 (75.9%)
Practicing social distancing by maintaining a 6-foot distance from others when in a public space	231 (93.2%)	196 (92%)	29 (100%)
In self-quarantine (not leaving the house at all) because of positive COVID-19 test or symptoms	2 (0.8%)	2 (0.9%)	0
In self-quarantine (not leaving the house at all) because of contact with someone who was infected with COVID-19	1 (0.4%)	1 (0.5%)	0
In self-quarantine (not leaving the house at all) because unsure of infection status	6 (2.4%)	4 (1.9%)	2 (6.9%)
Not making any changes to one’s daily life and routine	1 (0.4%)	1 (0.5%)	0
Other	81 (32.9%)	67 (31.6%)	12 (42.9%)
			
**COVID-19 symptoms, Care Seeking and Testing**			
>1 co-occurring COVID-19 symptom	96 (38.7%)	84 (39.4%)	9 (31%)
Sought care	43 (17.3%)	41 (19.2%)	1 (3.6%)
Hospitalized because of suspicion of COVID-19 or COVID-19 related illness	6 (2.5%)	5 (2.4%)	0
Tested for COVID-19	156 (63.2%)	137 (64.6%)	16 (55.2%)
Ever tested positive for COVID-19	28 (18.2%)	24 (19.7%)	4 (12.9%)
			
**COVID-19 Treatments**			
Lopinavir/ritonavir (Kaletra)	0	0	0
Hydroxychloroquine (Plaquenil)	5 (2.0%)	5 (2.3%)	0
Hydroxychloroguine (Plaquenil) with azithromycin (Zithromax, Z-pak)	6 (2.4%)	5 (2.3%)	0
Chloroquine	1 (0.4%)	1 (0.5%)	0
Ribavirin, also known as Moderiba or Rebetol	0	0	0
Remdesivir	1 (0.4%)	1 (0.5%)	0
Azithromycin	10 (4.0%)	10 (4.7%)	0
plasma transfusion/infusion	2 (0.8%)	1 (0.5%)	0
Aspirin	6 (2.4%)	5 (2.3%)	0
Other	23 (9.2%)	23 (10.8%)	0
			
**Increased use of pain medications**, N (%) yes			
Over the counter pain medications	55 (22.1%)	50 (23.4%)	2 (6.9%)
Prescription pain medications	19 (7.7%)	18 (8.5%)	1 (3.5%)

**Table 3.** Mental health during COVID-19 among clinical and non-clinical ED HCW in Brooklyn, New York.

	Total Mean (SD) or N (%)	Clinical HCW Mean (SD) or N (%)	Non-Clinical HCW Mean (SD) or N (%)	p-value
				
**Mental Health Scale Scores**				
Depression (CES-D)	11.8 (8.2)	12.5 (8.3)	8.0 (6.1)	0.008
Psychological distress (K10)	17.7 (8.4)	18.4 (8.8)	13.7 (4.1)	<0.0001
Perceived stress (PSS-4)	6.5 (3.4)	6.7 (3.4)	5.1 (2.9)	0.020
Post-traumatic stress disorder (PCL-5)	15.90 (17.4)	17.1 (17.9)	8.1 (10.7)	0.001
Anxiety (PROMIS®)	2.2 (2.2)	2.3 (2.2)	1.3 (1.6)	0.019
Resilience (BRCS)	11.1 (2.5)	11.0 (2.5)	11.8 (2.7)	0.141
				
**Clinically-relevant mental health burden**				
Depression (CES-D≥10)	113 (51.6%)	103 (53.9%)	10 (35.7%)	0.072
Psychological distress (K10≥20)	73 (32.9%)	69 (35.8%)	4 (13.8%)	0.019
Perceived stress (PSS-4≥6)	142 (61.2%)	129 (63.6%)	13 (44.8%)	0.053
Post-traumatic stress disorder (PCL-5≥32)	34 (16.3%)	33 (18.2%)	1 (3.6%)	0.064

**Table 4.** Odds of clinically-relevant mental health symptoms during COVID-19 in Emergency Department healthcare workers in Brooklyn, New York.

	Depression		Psychological Distress		Perceived Stress		Post-traumatic stress disorder	
Independent variable*	Odds Ratio (95%CI)	p-value	Odds Ratio (95%CI)	p-value	Odds Ratio (95%CI)	p-value	Odds Ratio (95%CI)	p-value
**Healthcare worker status**								
**Medical Doctor/Doctor of Osteopathy**	1.82 (0.66,5.04)	0.247	0.99 (0.30,3.20)	0.982	4.84 (1.82,12.86)	0.002	5.45 (0.52,57.30)	0.158
**Registered Nurse/Nurse Practitioner/physician assistant**	0.72 (0.24,2.18)	0.557	0.66 (0.18,2.44)	0.535	1.24 (0.44,3.47)	0.683	3.08 (0.28,34.44)	0.362
**Emergency Medical Technician**	1.39 (0.45,4.30)	0.565	1.14 (0.34,3.77)	0.832	1.54 (0.57,4.17)	0.392	15.49 (1.60,150.37)	0.018
**Non-clinical (ref)**	ref		ref		ref		ref	
**Age (years)**	1.08 (1.04,1.12)	0.0001	1.00 (0.96,1.04)	0.995	1.03 (0.99,1.06)	0.141	0.98 (0.93,1.03)	0.463
**Male (female ref)**	0.34 (0.15,0.76)	0.009	0.71 (0.30,1.66)	0.429	0.58 (0.28,1.23)	0.155	0.40 (0.12,1.32)	0.133
**No. of Covid-19-related home problems**	1.49 (1.02,2.17)	0.037	0.97 (0.64,1.46)	0.877	2.01 (1.34,3.02)	0.001	1.53 (0.92,2.54)	0.100
**Survey Date**								
**Survey date November/December**	0.52 (0.21,1.28)	0.155	0.53 (0.19,1.45)	0.214	2.30 (0.97,5.43)	0.057	0.51 (0.13,1.93)	0.319
**Survey date September/October**	ref		ref		ref		ref	
**Loneliness score**	2.09 (1.63,2.67)	<0.0001	2.20 (1.69,2.86)	<0.0001	1.68 (1.34,2.10)	<0.0001	1.69 (1.28,2.23)	0.0002
**Mental healthcare interruptions**								
**Interrupted mental healthcare**	0.36 (0.10,1.35)	0.129	0.29 (0.08,1.11)	0.071	0.33 (0.07,1.50)	0.151	0.67 (0.17,2.76)	0.584
**No interrupted mental healthcare**	ref		ref		ref		ref	
**Social Support**								
**≥4 people to talk to**	1.08 (0.30,3.82)	0.909	12.40 (2.30, 66.86)	0.003	1.70 (0.52, 5.58)	0.380	6.38 (0.92,44.21)	0.061
**2–3 people to talk to**	0.87 (0.32,2.35)	0.777	6.04 (1.30,28.05)	0.022	0.72 (0.29, 1.77)	0.477	1.59 (0.26,9.72)	0.619
**1 person to talk to**	0.94 (0.31,2.90)	0.9 19	6.31 (1.25,31.78)	0.026	0.46 (0.16,1.29)	0.138	1.82 (0.27,12.07)	0.537
**No one to talk to**	ref		ref		ref		ref	0.061
**SARS-CoV-2 Testing Status**								0.619
**SARS-CoV-2 (−)**	0.76 (0.35,1.63)	0.474	0.40 (0.17,0.94)	0.035	0.50 (0.24,1.04)	0.064	0.50 (0.18,1.43)	0.198
**SARS-CoV-2 (+)**	2.02 (0.56,7.23)	0.281	1.26 (0.34,4.72)	0.732	0.26 (0.08,0.84)	0.025	0.32 (0.05,1.97)	0.218
**Not tested for SARS-CoV-2**	ref		ref		ref		ref	0.218
**Number of respondents**	**207**		**210**		**219**		**195**	

* 30.5% (N=75) Medical Doctor/Doctor of Osteopathy, 23.6% (N=58) Registered Nurse/Nurse Practitioner/physician assistant, 25.2% (N=62)

Emergency Medical Technician, 20.7% (N=51) non-clinical; 51.8% (N=127) men, 48.2% (N=118) women; 78.5% (N=194) September/October

Survey date, 21.46% (N=21.5%) November/December survey date; 90.8% (N=226) no interrupted mental healthcare; 19.3% (N=47) no one to talk to, 21.0% (N=51) 1 person to talk to, 39.1% (N=95) 2–3 people to talk to, 20.6% (N=50) ≥4 people to talk to; 50.83% (N=123) SARS-CoV-2 (−), 11.6% (N=28) SARS-CoV-2 (+), 37.6% (N=91) not tested.

**Table 5.** Multivariable linear regression analyses predicting mental health outcomes during COVID-19 in ED HCW in Brooklyn, New York.k

	Mental Health Outcome									
	Depression		Psychological Distress		Perceived Stress		Post-traumatic stress disorder		Resilience	
Independent variable^*^	beta	P-value	beta	P-value	beta	P-value	beta	P-value	beta	P-value
**HCW status**										
Medical Doctor/Doctor of Osteopathy	1.361	0.294	1.491	0.256	1.436	0.010	2.803	0.326	0.547	0.259
Registered Nurse/Nurse/Practitioner/Physician Assistant	1.334	0.349	0.162	0.908	0.648	0.281	1.883	0.537	0.551	0.287
Emergency Medical Technician	1.705	0.217	3.690	0.007	0.699	0.230	7.501	0.012	0.782	0.124
Non-clinical (ref)	0	.	0	.	0	.	0	.	0	.
Age (years)	0.1 15	0.010	0.013	0.776	0.021	0.260	0.054	0.571	0.013	0.420
Male (female ref)	1.810	0.063	1.979	0.043	0.869	0.035	4.306	0.048	0.379	0.296
Number of Covid-19-related home problems	0.994	0.035	0.615	0.2	0.694	0.001	2.133	0.042	0.150	0.400
Survey date Nov/Dec	1.861	0.096	0.357	0.747	0.298	0.532	2.896	0.248	0.441	0.285
Survey date Sep/Oct (ref)	0	.	0	.	0	.	0	.	0	.
Loneliness score	2.169	<0.0001	2.197	<0.0001	0.617	<0.0001	4.095	<0.0001	0.109	0.251
Interrupted mental healthcare (yes)	1.931	0.221	3.452	0.036	1.047	0.133	5.317	0.135	0.166	0.785
**Social Support**										
≥4 people to talk to	2.090	0.180	3.674	0.018	1.443	0.028	6.971	0.038	1.518	0.009
2–3 people to talk to	0.627	0.622	1.062	0.403	0.316	0.555	1.892	0.481	0.447	0.343
1 person to talk to	0.518	0.714	1.289	0.371	1.024	0.091	1.255	0.681	0.312	0.553
No one to talk to (ref)	0	.	0	.	0	.	0	.	0	.
**SARS-CoV-2 Testing Status**										
SARS-CoV-2 (−)	0.892	0.360	1.856	0.054	1.111	0.008	1.483	0.480	0.806	0.027
SARS-CoV-2 (+)	0.971	0.520	1.136	0.474	1.649	0.012	4.386	0.211	0.082	0.888
Not tested for SARS-CoV-2 (ref)	0	.	0	.	0	.	0	.	0	.
**Number of respondents**	211		215		223		201		224	

* 30.5% (N=75) Medical Doctor/Doctor of Osteopathy, 23.6% (N=58) Registered Nurse/Nurse Practitioner/physician assistant, 25.2% (N=62) Emergency Medical Technician, 20.7% (N=51) non-clinical; 51.8% (N=127) men, 48.2% (N=118) women; 78.5% (N=194) September/October Survey date, 1.46% (N=21.5%) November/December survey date; 90.8% (N=226) no interrupted mental healthcare; 19.3% (N=47) no one to talk to, 21.0% (N=51) 1 person to talk to, 39.1% (N=95) 2–3 people to talk to, 20.6% (N=50) >4 people to talk to; 50.83% (N=123) SARS-CoV-2 (−), 11.6% (N=28) SARS-CoV-2 (+), 37.6% (N=91) not tested
